# Health risks of cocaine adulteration: local anesthetics as modulators of monoamine and organic cation transporters

**DOI:** 10.3389/fphar.2025.1699035

**Published:** 2025-11-19

**Authors:** Oliver Kudlacek, Niklas Senning, Alexandra Karden, Iina Ludwig, Julia Bicher, Fatemeh Kooti, Marion Holy, Thomas Stockner, Anton Luf, Harald H. Sitte

**Affiliations:** 1 Institute of Pharmacology, Center for Physiology and Pharmacology, Medical University of Vienna, Vienna, Austria; 2 checkit!-Suchthilfe Wien gGmbH (Vienna Addiction Services), Vienna, Austria; 3 Department of Social and Preventive Medicine, Center for Public Health, Medical University of Vienna, Vienna, Austria; 4 Clinical Department of Laboratory Medicine, Medical University of Vienna, Vienna, Austria; 5 Hourani Center for Applied Scientific Research, Al-Ahliyya Amman University, Amman, Jordan; 6 Center for Addiction Research and Science, Medical University of Vienna, Vienna, Austria

**Keywords:** local anesthetics, cocaine adulteration, monoamine transporters, OCT inhibition, harm reduction

## Abstract

**Introduction:**

Local anesthetics (LAs) are frequently used as adulterants in cocaine sold on the illicit market, sometimes in higher quantities than cocaine itself. These agents can mimic cocaine’s anesthetic effect, masking the products reduced purity. While reports suggest that LAs influence monoaminergic neurotransmission, systematic evidence remains limited. We examined three LAs commonly detected in cocaine samples submitted for drug checking from Vienna, Austriaprocaine, benzocaine, and lidocainefor their activity on uptake‐1 monoamine transporters (DAT, NET, SERT) and uptake‐2 organic cation transporters (OCT13).

**Methods and Results:**

Transporter activity was measured in vitro, and computational docking was applied to explore molecular interactions with atomistic detail. Procaine and benzocaine inhibited DAT and NET at physiologically relevant concentrations, whereas neither compound affected SERT. Procaine also inhibited OCT1 and OCT2 with affinities comparable to or greater than cocaine, while benzocaine exhibited no OCT activity. Lidocaine had no significant effect on any transporter. Docking studies confirmed procaine binding within the DAT substrate pocket, consistent with its inhibitory profile.

**Discussion:**

Although LAs modulate uptake-1 and uptake-2 transporters, their actions are insufficient to replicate cocaine’s psychoactive effects. However, their impact on OCTs indicates potential health risks, highlighting the importance of accessible drug checking services for harm reduction.

## Introduction

Cocaine is one of the most widely used illicit drugs worldwide (World Drug Report^1^). It is an alkaloid found in *Erythroxylum Coca* and other species of the genus *Erythroxylum*. The isolation and development of cocaine as the first local anesthetic with central nervous system activity is historically linked to the Novara expedition in the 19th century (Niemann, 1860). Since then, cocaine has been employed as a local anesthetic (Markel, 2011), with self-administration experiments revealing both the mechanism underlying its anesthetic action and its psychoactive and addictive properties.

Local anesthetic effects are mediated by cocaine’s ability to block voltage-gated sodium channels (Roque Bravo et al., 2022). Its psychological effects, however, primarily result from inhibition of the dopamine transporter (DAT), which elevates extraneuronal dopamine levels and, subsequently, activates pre- and postsynaptic dopamine receptors, as well as from competitive inhibition of noradrenaline (NET) and serotonin (SERT)— -transporter collectively referred to as uptake-1 transporters (Buchanan et al., 2021). Cocaine’s euphoric effects drive the development of chronic abuse, which in turn disrupts neurotransmitter and neuroendocrine balance. Research suggests that excessive stimulation of dopamine-producing neurons may contribute to reduced dopamine availability over time (Dackis and Gold, 1985).

Substantial inhibitory potency of cocaine has recently been described at organic cation transporters (OCT) of the SLC22 family OCT1 and OCT2 but not OCT3 (Angenoorth et al., 2021). OCTs have been characterized as uptake-2 transporters or low-affinity, high-capacity transporters that operate in an alternating access mode, using only the substrate gradient as an energy source, unlike uptake-1 monoamine transporters (Koepsell, 2020). OCT1 and OCT3 are widely expressed, with OCT1 prevalent in the liver and OCT3 in the heart and nervous system, whereas OCT2 is mainly localized to the kidney and, to a lesser extent, other tissues (Koepsell, 2020). OCT3, in particular, plays crucial roles in the uptake of dopamine (DA) and noradrenaline (NA) in the brain, and has been implicated in psychiatric diseases such as major depression, with OCT3 knockout mice displaying increased anxiety-like traits (Daws, 2021).

Due to its highly addictive potential, cocaine was reclassified as an illicit drug (Goldstein et al., 2009) and withdrawn from the legal market. Without regulatory oversight, cocaine nowadays is frequently adulterated with “cutting agents”, which include bulking agents or pharmacologically active substances designed to increase product volume and dealer profit. This phenomenon is not only found for cocaine, but also for other illicitly purchased drugs, including opioids (Singh et al., 2020).

To help combat unintentional poisoning, users in Vienna, Austria, have been able to submit illicitly sourced drugs for anonymous chemical analysis at the drug checking service checkit!. Operational since 1997, checkit! collates information on the substances circulating in the Viennese illicit drug market. Over the past decades, systematic analyses of illicit cocaine have revealed that the market is highly dynamic and characterized by the pervasive presence of adulterants and additives in a large number of samples. These adulterants vary from analgesics and local anesthetics (LA) to unexpected compounds like the anthelmintic drug levamisole (Hofmaier et al., 2014).

The main criteria for selecting adulterants are that they are inexpensive and readily available, such as inert bulking agents, and that they mimic cocaine’s sensory or pharmacological effects to mask reduced purity, as is the case with LAs (Kudlacek et al., 2017). Critically, illicit cocaine mixtures are often required to pass informal “quality checks” by users, who may assess its melting behavior, taste, or characteristic oral numbing sensation. Partial mimicry of cocaine’s psychoactive effects is also desirable, since experienced users generally know what to expect. Local anesthetics reproduce cocaine’s numbing effect when applied to the tongue or gingiva. This corresponds to cocaine’s well-known sensory signature, first described by Albert Niemann in 1860: “*a bitter taste, leaves a strange numb feeling on the tongue, followed by a sensation of coldness in the mouth”* (Niemann, 1860).

While local anesthetics are added to mimic cocaine’s characteristic sensory effects, other adulterants are incorporated for entirely different reasons. One of the most notable examples is levamisole (Midthun et al., 2021), its use as a cocaine adulterant stems, in part, from its metabolism to aminorex, which exerts amphetamine-like properties (Hofmaier et al., 2014).

Although structurally similar to cocaine, newer LAs were developed to retain its local anesthetic effects without exerting any psychoactive effects. Nevertheless, several reports have since linked LAs used in the clinic to central nervous system actions (Adinoff et al., 1998; Sato et al., 2000).

In the present work, we systematically examined LAs identified as cocaine adulterants in Vienna, Austria, for their actions on clinically relevant targets of cocaine: the high-affinity, low-capacity re-uptake transporters for dopamine, noradrenaline and serotonin (DAT, NET, and SERT; uptake-1 transporters) and the low-affinity, high-capacity transporters OCT1–OCT3 (uptake-2 transporters). Our results reveal a nuanced profile: among the LAs derived from cocaine, the two ester-type compounds—procaine and benzocaine—inhibited DAT and NET but not SERT, whereas the amide-type LA, lidocaine, showed no significant effect on these transporters. Effects on OCTs were more variable: procaine inhibited OCT1 and OCT2 but not OCT3; benzocaine exhibited no inhibitory activity on any of the OCTs; and lidocaine had a negligible effect.

## Materials and methods

### Drugs and reagents

Unless otherwise specified, chemicals were obtained from Sigma Aldrich (St Louis, MO, United States). Procaine was purchased from Merck (Darmstadt, Germany), lidocaine from Thermo Fisher Scientific (Waltham, MA, United States), and benzocaine (Ethyl 4-Aminobenzoate) from TCI Deutschland (Eschborn, Germany). D22 was obtained from SYNTHON Chemicals (Bitterfeld-Wolfen, Germany) and paroxetine hydrochloride from abcr (Karlsruhe, Germany). Cell culture supplies were provided by Capricorn Scientific (Ebsdorfergrund, Germany). Radiolabeled tracers were provided from Revvity (Waltham, MA, United States), including [^3^H]-serotonin ([^3^H]-5-HT), and [^3^H]1-methyl-4-phenylpyridinium ([^3^H]MPP^+^).

### Cell culture

Human embryonic kidney (HEK293) cells stably expressing the human isoform (h) of the transporters were grown at 37 °C in a humidified atmosphere of 5% CO_2_, described in detail in Mayer et al. (2018). Cells were cultured in Dulbecco’s modified Eagle’s medium (DMEM) supplemented with 10% fetal bovine serum and 250 μg/mL geneticin to maintain selection pressure.

### Uptake inhibition assay

HEK293 cells heterologously expressing the respective transporters were seeded onto poly-D-lysine (PDL)-coated 96-well plates at a density of ∼36,000 cells per well, 1 day before experiments. For the experiment, medium was replaced with 200 µL Krebs HEPES buffer (KHB; 120 mM NaCl, 3 mM KCl, 2 mM CaCl_2_·2H_2_O, 2 mM MgCl_2_·6H_2_O, 20 mM D-glucose, pH 7.3). Test compounds were dissolved in Milli-Q water or dimethyl sulfoxide (DMSO) at 10–100 mM stock concentrations. Cells were preincubated for 5 min with the compound of interest (diluted in KHB to the indicated concentrations). The preincubation solution was then replaced with 50 µL/well of diluted compound together with tritiated substrate (DAT, OCT1, OCT2, OCT3: 0.05 μM [^3^H]MPP^+^; NET: 0.02 μM [^3^H]MPP^+^; SERT: 0.1 μM [^3^H]5-HT) for 1 min (SERT) or 3 min (DAT, NET, OCT1– OCT3). After the incubation, uptake was terminated by rapidly washing cell with 200 μL KHB, followed by addition of 200 μL Ultima Gold^™^ XR scintillation cocktail (PerkinElmer, MA, United States) to each well. Radioactive uptake was quantified by liquid scintillation counting using a Wallac 1450 MicroBeta TriLux counter (PerkinElmer; GMI, Ramsey, MN, United States). Uptake in the absence of inhibitor was defined as 100%, while uptake in presence of a reference inhibitor (hDAT, hNET: 50 μM GBR12909; hSERT: 3 μM paroxetine; OCT1–OCT3: 100 µM D22) was defined as nonspecific uptake (0%) and subtracted from all values.

### Superfusion assay

One day prior to the experiment, HEK293 cells expressing DAT were seeded at a density of 100,000 cells per channel into 6-channel flow slides (ibidi, Gräfelfing, Germany). On the day of the experiment, cells were preloaded with 0.05 μM [^3^H]MPP^+^ for 20 min at 37 °C in 5% CO_2_. Slides were then transferred to the superfusion apparatus, as previously described (Brugnoli et al., 2023). Cells were superfused with KHB for 15 min at a constant flow of 0.5 mL/min. Fractions were collected every 2 min into 8 mL vials containing 2 mL scintillation cocktail. After collection of three baseline fractions (KHB), four additional basal release fractions were obtained in the presence or absence of 10 µM monensin. Subsequently, the test compound (10 µM (*S*)-amphetamine, 20 µM procaine, or 0.5 µM cocaine) was perfused, and five fractions were collected. To assess residual radioactivity, cells were lysed with 1% sodium dodecyl sulfate (SDS), and three final fractions were collected. Radioactivity in each fraction was expressed as a percentage of the total tritiated substrate present at the start of that fraction.

### In silico docking

In silico docking was performed using AutoDock Vina 1.2.0. Protein Data Bank (PDB) files for the transporters and ligands were preprocessed in UCSF Chimera (Pettersen et al., 2004). The structures were then converted into PDBQT format using Meeko (https://github.com/forlilab/meeko). Docking simulations were run with an exhaustiveness setting of 32, using a grid box optimized for ligand positioning and orientation. Ligand-protein interaction fingerprints of selected docking poses were subsequently generated with ProLIF (Bouysset and Fiorucci, 2021).

### Drug checking data from checkit!

checkit! is a scientific cooperation between the Viennese Addiction Services and the Medical University of Vienna and offers its services anonymous and free of charge to users of psychoactive substances. All cocaine samples between 2015 and 2024 were submitted for drug checking either on-site at music events and from 2019 onwards, additionally at the drop-in center or through selected pharmacies in Vienna. For quantitative and qualitative chemical analysis, service users submitted 5–10 mg of cocaine powder. The chemical analysis was carried out using UHPLC-DAD-MS (Ultra-high-performance liquid chromatography - diode array detection - mass spectrometry) employing a Dionex Ultimate 3000 RS (Thermo Fisher Scientific, MA, United States) chromatography system coupled with a Shimadzu SPD M20A diode array detector (Shimadzu, Kyoto, Japan) for quantification and a Thermo Scientific Velos pro ion trap mass spectrometer (Thermo Electron Corporation, CA, United States) for unambiguous identification. The exact analytical protocol for sample preparation, identification and quantification can be found elsewhere (Pulver et al., 2023). For quantification of cocaine and procaine, calibration solutions and quality control samples were prepared using the corresponding hydrochloride salts.

## Results

### Patterns of cocaine adulteration in Vienna (2012–2024)

**FIGURE 1:**
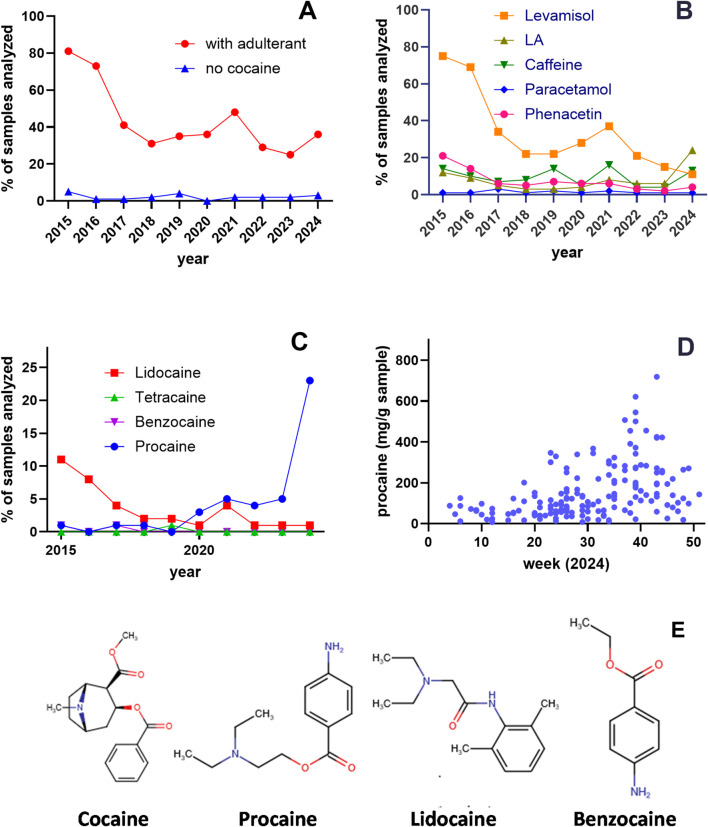
Adulterants in cocaine samples in Vienna, Austria (2015–2024). **(A)** Analysis of 3,829 samples submitted as cocaine to the “checkit!” drug checking service. The proportion of samples containing adulterants decreased from 81% in 2015 to 36% in 2024 (red circles), while cocaine-free samples remained consistently low (blue triangles; 0%–5%). **(B)** Percentage of adulterants detected across analyzed samples. Levamisole (orange squares) decreased from 75% in 2015 to 11% in 2024, whereas local anesthetics (LAs; black triangles) increased from 15% in 2015 to 24% in 2024. **(C)** Year-by-year prevalence of samples containing lidocaine, benzocaine, and procaine. Samples adulterated with procaine (blue circles) increased from 1% in 2015 to 24% in 2024, while lidocaine (red squares) decreased from 11% in 2015 to 1% in 2024; other LAs were rarely detected. **(D)** Procaine content (mg/g) in procaine-positive samples analyzed by CheckIt! in 2024. **(E)** Chemical structures of cocaine, procaine, lidocaine, and benzocaine. Structures were drawn using Chemicalize (ChemAxon; https://chemicalize.com/, accessed July 2025).

In 2012, our analysis identified levamisole as the predominant adulterant in cocaine sold in Vienna (Hofmaier et al., 2014). Over the following decade (2015–2024), the overall pattern of cutting agents in cocaine samples submitted to the Viennese drug checking service shifted. The proportion of cocaine-free samples remained consistently low, ranging from 0% in 2020 to a maximum of 5% in 2015. By contrast, the proportion of samples containing pure cocaine increased markedly: in 2015, only 13% of samples contained unadulterated cocaine, whereas from 2017 onward, at least 50% of samples were free from additives (mean 63.13% ± 2.58) (Figure 1A).

Although the library of adulterants remained largely unchanged, their relative frequencies varied over time. Levamisole-adulterated samples decreased sharply from 75% in 2015 to 11% in 2024. Phenacetin was present in 21% of samples in 2015 but fell to <10% after 2017. Paracetamol (acetaminophen) remained consistently low throughout the study period (mean 1.44% ± 0.22), and caffeine showed fluctuating prevalence, ranging from 4% to 16% (mean 9.5% ± 1.43) (Figure 1B).

LAs were consistently detected throughout the survey period. In 2015, 12% of samples contained an LA, with lidocaine being the most common (Figure 1C). Although LAs prevalence decreased in subsequent years, by 2024 the percentage of LA-adulterated cocaine samples had suddenly risen again to 24% with procaine as the predominant agent. Of the 168 procaine-positive samples analyzed in 2024, the mean procaine content was 153 mg/g (maximum 718 mg/g) (Figure 1D).

The choice of adulterants has long been the subject of speculation. However, explanations for the use of other adulterants remain limited. To address this gap, we systematically screened adulterants found in cocaine samples for pharmacological activity at key psychostimulant targets—the monoamine transporters DAT, SERT and NET.

### Inhibitory profile of adulterants at monoamine reuptake transporters

**FIGURE 2:**
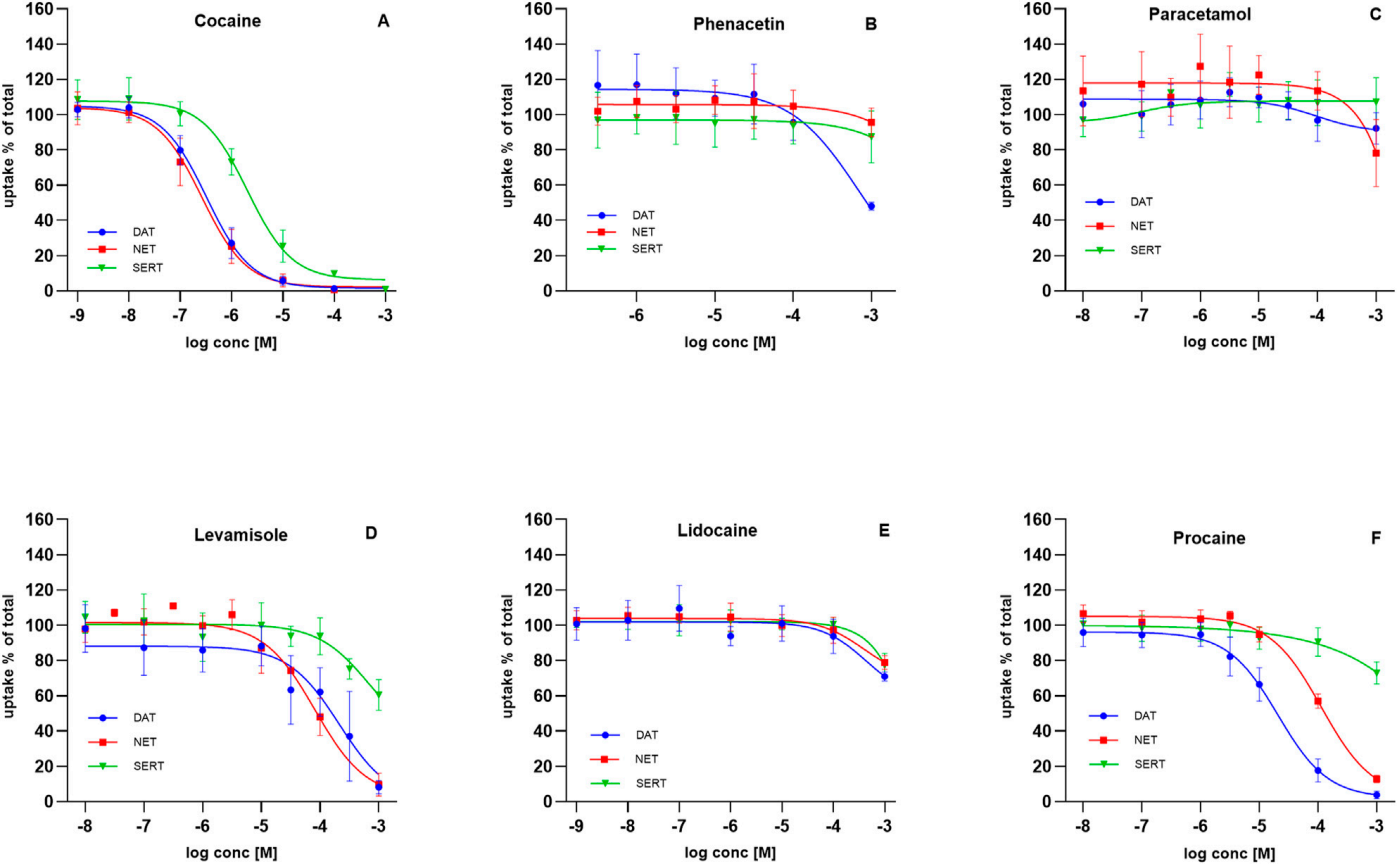
Effect of adulterants on substrate uptake at DAT, NET, and SERT. HEK293 cells heterologously expressing human DAT, NET or SERT were preincubated with the indicated compounds, followed by the addition of radiolabeled substrates: 0.05 µM [^3^H]MPP^+^ for DAT (blue circles), 0.02 µM [^3^H]MPP^+^ for NET (red squares), and 0.1 µM [^3^H]5-HT for SERT (green triangles). Uptake inhibited curves are shown for: **(A)** cocaine, **(B)** phenacetin, **(C)** paracetamol, **(D)** levamisole, **(E)** lidocaine, and **(F)** procaine. Uptake is expressed as a percentage of control (uptake in the absence of test compound). Data represent mean ± SD of ≥3 independent experiments.

Illicit market cocaine is commonly adulterated with levamisole, phenacetin, paracetamol, and various LAs (Kudlacek et al., 2017; Morelato et al., 2019; Hesse et al., 2021). Effective adulterants are typically inexpensive and difficult for users to distinguish from cocaine (Kudlacek et al., 2017; Knuth et al., 2018). To determine whether adulterants share pharmacological properties with cocaine, we compared their inhibitory activity at monoamine transporters with that of cocaine (Figure 2).

Cocaine potently blocked monoamine reuptake in the high nanomolar to low micromolar range. Inhibition of DAT and NET occurred at similar potencies (IC_50_ = 0.27–0.39 µM for DAT; 0.20–0.36 µM for NET). SERT inhibition was weaker, with IC_50_ values ranging from 1.46 to 2.84 µM (Figure 2A).

Phenacetin and paracetamol displayed no measurable inhibitory activity at DAT, NET, or SERT across the tested range (Figures 2B,C). Levamisole, in contrast, inhibited uptake at higher concentrations, with IC_50_ values of 81–316 µM (DAT), 60–106 µM (NET), and 235–1881 µM (SERT) (Figure 2D), consistent with previous reports (Hofmaier et al., 2014).

Local anesthetics were of particular interest given their prevalence in collected samples. Since cocaine blocks not only monoamine transporters but also voltage-gated sodium channels, we investigated whether LAs serve solely to mimic cocaine’s sensory numbing effect or whether they also act at monoamine transporters. Lidocaine displayed no inhibitory potency at DAT, NET, or SERT (Figure 2E). In contrast, procaine showed selective activity: DAT inhibition occurred with an IC_50_ of 15–29 µM and NET inhibition with an IC_50_ of 88–146 μM, whereas SERT was unaffected at sub-millimolar concentrations (Figure 2F).

### Local anesthetics at Uptake-2 transporters OCT1–3

**FIGURE 3:**
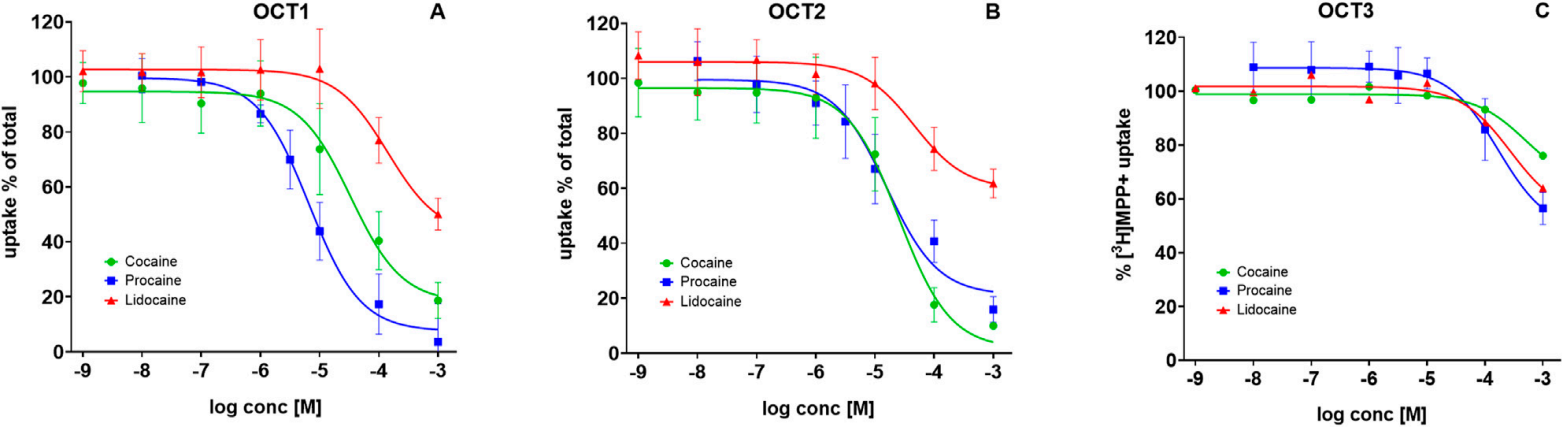
Inhibition of substrate uptake by local anesthetics at OCT1–3. HEK293 cells heterologously expressing human: **(A)** OCT1, **(B)** OCT2, or **(C)** OCT3, were preincubated with the indicated compounds, followed by addition of 0.05 µM [^3^H]MPP^+^. Uptake inhibition curves are shown for cocaine (green circles), procaine (blue squares), and lidocaine (green triangles). Uptake is expressed as a percentage of control (uptake in the absence of test compound). Data represent mean ± SD of ≥3 independent experiments.

We previously reported that cocaine inhibits OCT1 and OCT2, but not OCT3 (Angenoorth et al., 2021). To test whether LAs from cocaine samples differ in their potency, we compared procaine and lidocaine with cocaine. The variability was striking. Cocaine inhibited OCT1-mediated [^3^H]MPP^+^ transport with an IC_50_ of 19–57 μM, whereas procaine was more potent (IC_50_ = 5–9 µM) and lidocaine less potent (IC_50_ = 79–252 µM) (Figure 3A). At OCT2, cocaine blocked uptake with an IC_50_ of 15–42 μM, and procaine showed similar potency (IC_50_ = 10–24 µM). Lidocaine, however, reduced uptake only partially (to 53%–66%) with an IC_50_ of 23–90 µM (Figure 3B). In contrast, none of the compounds—cocaine, procaine, or lidocaine—substantially inhibited OCT3-mediated transport except at near-millimolar concentrations (Figure 3C).

### Procaine does not induce substrate efflux via DAT

Previous studies have shown that procaine administration elevates the concentration of dopamine in the brain of rodents (Woodward et al., 1995). However, in native tissue it is difficult to distinguish between amphetamine-like releasing activity and simple uptake inhibition. A release assay in substrate-preloaded cells is the most effective method to discriminate between these mechanisms: addition of a releasing agent evokes substrate efflux, which is further enhanced by the Na^+^-ionophore monensin (Scholze et al., 2000).

**FIGURE 4:**
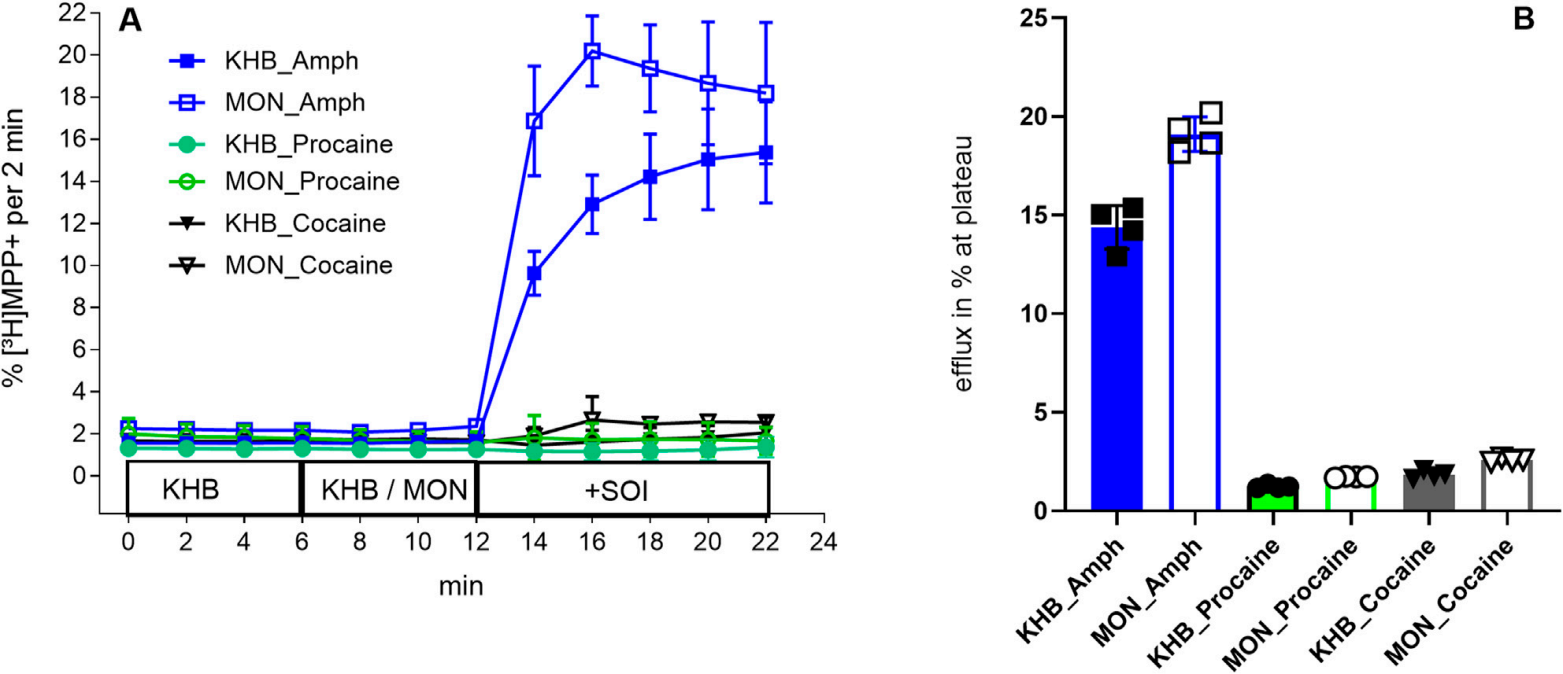
Neither Cocaine nor Procaine evoke substrate efflux via DAT. HEK293 cells heterologously expressing human DAT were preloaded with 0.05 µM [^3^H]MPP^+^ for 20 min with and then superfused with Krebs HEPES buffer (KHB) for 40 min to reach stable efflux baseline. Fractions were collected every 2 min. **(A)** Time course of [^3^H]MPP^+^ efflux. Cells were exposed at 6 min to KHB (filled symbols) or 10 µM monensin (open symbols), followed at 12 min by 10 µM (*S*)-amphetamine (blue squares), 20 µM procaine (green circles), or 0.5 µM cocaine (black triangles). At 22 min, cells were lysed with 1% SDS to determine residual substrate. **(B)** Mean % [^3^H]MPP^+^ released at plateau for KHB- and MON-treated conditions with (*S*)-amphetamine, procaine, or cocaine. Symbols above each bar (filled/open squares, circles, or triangles) represent efflux in % at plateau of single experiments. Only (*S*)-amphetamine evoked significant release (14.39% ± 2.18 per 2 min), which was further enhanced by monensin (19.10% ± 2.50 per 2 min). Neither cocaine nor procaine elicited substrate release in both the absence and presence of monensin. Data are mean ± SD of ≥3 independent experiments.

To test whether procaine elicits transporter-mediated efflux, we compared its effects with those of amphetamine and cocaine. As expected (Scholze et al., 2002), (*S*)-amphetamine (10 µM) induced substrate release from human DAT (hDAT) expressing HEK293 cells preloaded with MPP^+^ (filled blue squares/bar), and this effect was potentiated by monensin (10 μM; open blue squares/bar). In contrast, neither cocaine (black triangles) nor procaine (green circles) produced a similar efflux response (Figure 4). Thus, similar to cocaine, we inferred that procaine inhibits DAT-mediated uptake but does not induce amphetamine-like substrate release.

### Benzocaine as an ester-type local anesthetic adulterant

Procaine displayed at least some transporter-inhibitory properties aligned with cocaine, whereas lidocaine was largely inactive at monoamine transporters. A key difference between these compounds is their chemical class: procaine, like cocaine, is an ester-type LA, while lidocaine is an amide-type. Both classes block voltage-gated sodium channels, but they differ in their ability to interfere with monoaminergic neurotransmission (Dorris and Gage, 1980). Our findings therefore support a pharmacological distinction between ester- and amide-type LAs.

**FIGURE 5:**
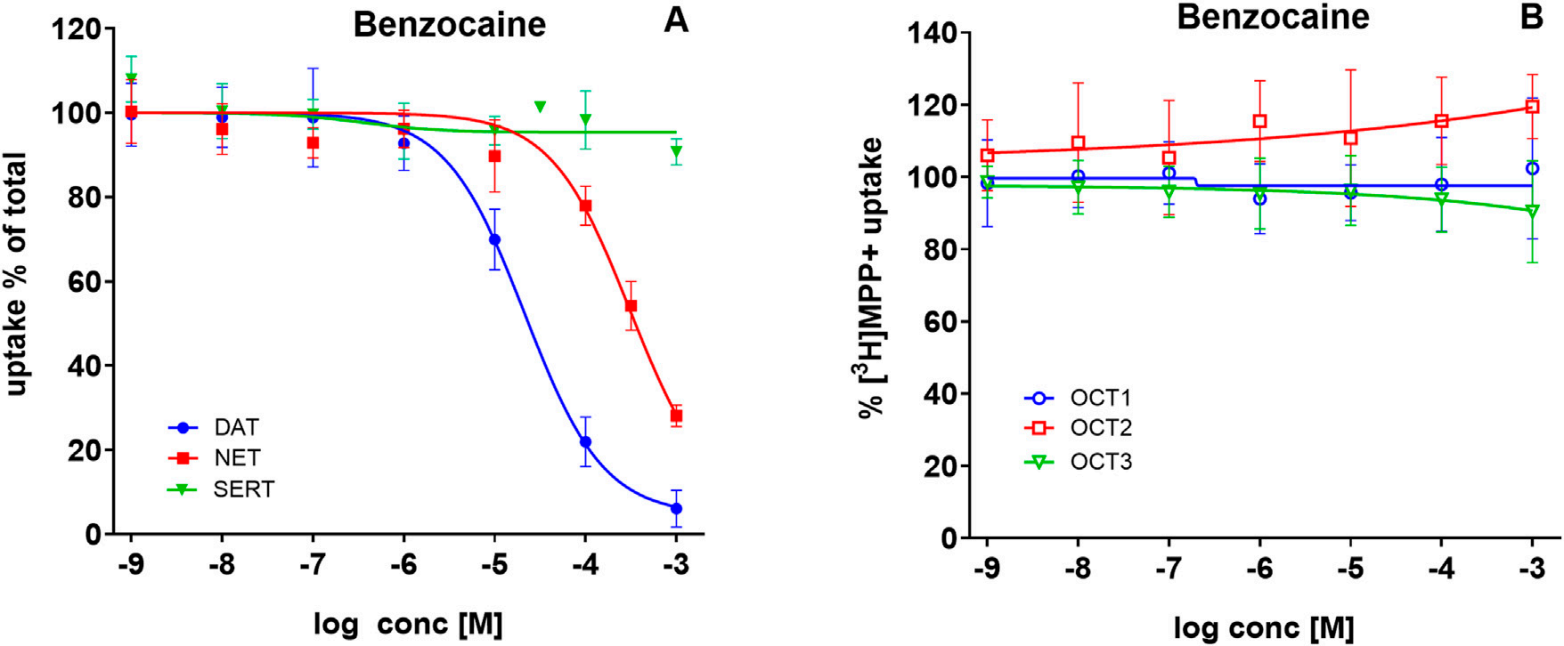
Benzocaine inhibits uptake via DAT and NET, but not SERT or OCT1–3. HEK293 cells heterologously expressing human DAT, NET, SERT, OCT1, OCT2, or OCT3 were incubated with increasing concentrations of benzocaine, followed by the addition of radiolabeled substrates. **(A)** Uptake of 0.05 µM [^3^H]MPP^+^ via DAT (blue circles), 0.02 µM [^3^H]MPP^+^ via NET (red squares), and 0.1 µM [^3^H]5-HT via SERT (green triangles) in the absence or presence of benzocaine. **(B)** Uptake of 0.05 µM [^3^H]MPP^+^ via OCT1 (open blue circles), OCT2 (open red squares), and OCT3 (open green triangles), in the absence or presence of benzocaine. Uptake is expressed as a percentage of control (uptake in the absence of benzocaine). Data represent mean ± SD of ≥3 independent experiments.

To test whether this property generalizes to other ester-type anesthetics, we examined benzocaine, another common cocaine adulterant. Benzocaine inhibited DAT-mediated uptake with IC_50_ values of 17–27 µM (Figure 5A, filled blue circles) in a range comparable to procaine. In contrast, its potency at NET was much lower (IC_50_ values = 215–444 μM; Figure 5A, filled red squares), approximately one order of magnitude weaker than procaine. As with procaine, benzocaine displayed no inhibitory activity at SERT (Figure 5A, green triangles). Furthermore, benzocaine failed to inhibit [^3^H]MPP^+^ transport in HEK293 cells via OCT1–3 (Figure 5B), in contrast to both procaine and cocaine.

### Computational pharmacology

**FIGURE 6:**
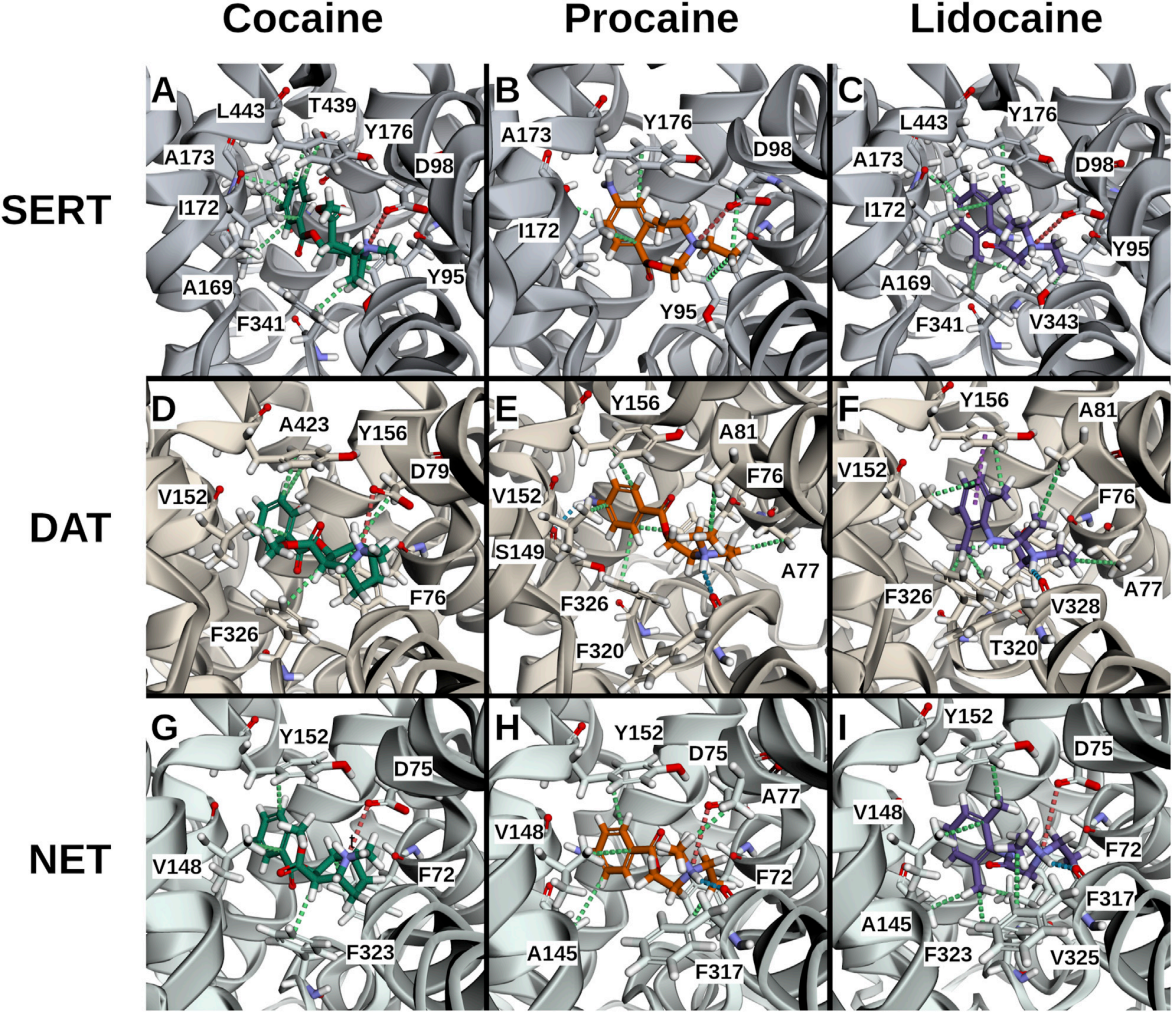
3D interaction plots of cocaine, procaine, and lidocaine in selected poses. Representative docking poses are shown for cocaine (**(A,D,G)**; poses 6, 0, 2), procaine (**(B,E,H)**; poses 1, 1, 4), and lidocaine (**(C,F,I)**; poses 1, 5, 0) bound to SERT **(A–C)**, DAT **(D–F)**, and NET **(G–I)**. Ligands are shown indeap teal (cocaine), vivid orange (procaine) and medium violet (lidocaine), transporters in blue-grey (SERT), warm brown (DAT) and teal (NET) respectively. Predicted interactions are depicted as dotted lines: hydrophobic (green), cationic (red), hydrogen bonds (blue), and π–π stacking (purple). Across all three transporters, ligands exhibited similar hydrophobic interactions from the sides (SERT: I172l; DAT: V152; NET: V148), from beneath (SERT: Y95; DAT: F76; NET: F72), and from above (SERT: Y176; DAT: Y156; NET: Y152), as well as ionic interactions between the ligand’s cationic ammonium and the anionic aspartates (SERT: D98; DAT: D79; NET: D75). Distinct preferences were also observed: cocaine favored hydrophobic interactions from beneath (SERT: F341; DAT: F326; NET: F323) and towards the rear (SERT: T439/L443; DAT: A423), whereas procaine and lidocaine favored hydrogen bonds involving their head groups at the front (DAT: F320; NET: F317). Lidocaine’s ortho-methyl ring substitution enabled additional hydrophobic interactions from beneath (SERT: V343; DAT: V328; NET: V325).

Cocaine, procaine, and benzocaine are ester-type LAs, whereas lidocaine is an amide-type. Their divergent inhibitory potencies at monoamine reuptake transporters (Figure 2) prompted us to examine binding interactions by docking these compounds (using cocaine as the parent substance) into the central binding site (S1) of cryo-EM structures of SERT [PDB ID: 7LIA], DAT [PDB ID: 9EO4], and NET [PDB ID: 8Y92]. Docking poses were consistent across the three transporters and, within experimental uncertainty, reproduced the experimentally determined cocaine-DAT complex (Nielsen et al., 2024), lending confidence to the predictions (Figure 6).

**FIGURE 7:**
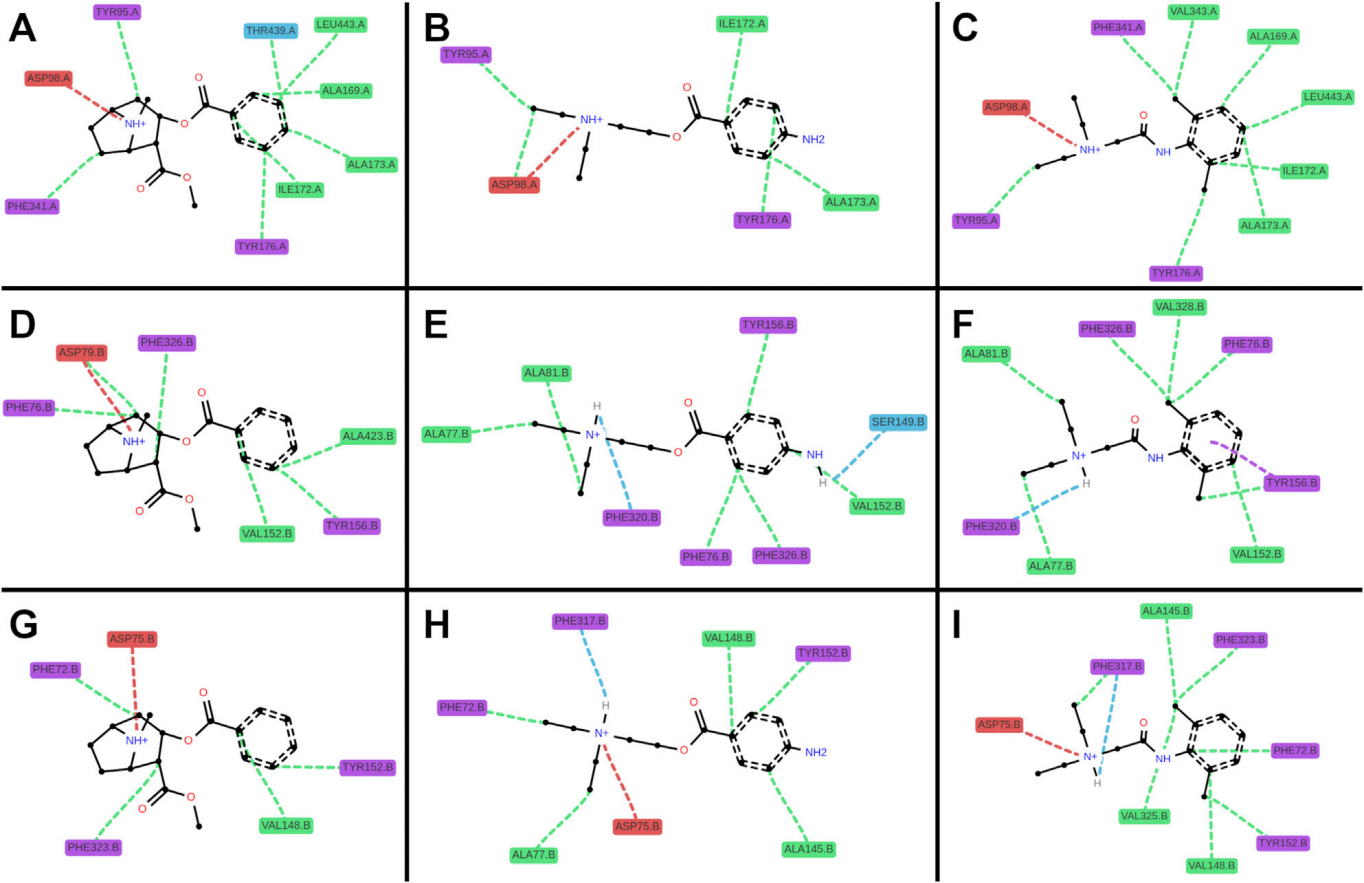
2D interaction plots of cocaine, procaine, and lidocaine in SERT, DAT, and NET. Representative docking poses are shown for cocaine (**(A,D,G)**; poses 6, 0, 2), procaine (**(B,E,H)**; poses 1, 1, 4), and lidocaine (**(C,F,I)**; poses 1, 5, 0) involving single amino acid interactions with SERT **(A–C)**, DAT **(D–F)**, and NET **(G–I)**. Colors denote amino acid classes: aliphatic (green), acidic (red), aromatic (purple), and polar (blue). Interactions are depicted as dotted lines: hydrophobic (green), cationic (red), hydrogen bonds (blue), and π–π stacking (purple).

All three ligands carry a positively charged amino group. As in known transporter-ligands complexes, this cation was positioned near the conserved aspartate in TM1 (D98 in SERT, D79 in DAT, D75 in NET) and the helical dipole of TM6a (Figure 6). ProLIF 2D fingerprint analysis confirmed these interactions by finding the ammonium cation interacting with the aspartate, with the phenylalanine (F335 in SERT, F320 in DAT, F317 in NET) of TM6a, or with both. Notably, ionic interactions persist even when slightly beyond the 4.5 Å cut-off applied in the ProLIF analysis due to the long-range nature of electrostatic interactions (Figure 7). Each ligand also positioned its aromatic ring (Figure 1E) in sub-pocket B of the transporter, where it engaged in stabilizing hydrophobic interactions with aliphatic and aromatic residues of TM3 and TM8. Cocaine, being slightly bulkier due to its additional methyl ester group (Figure 1E), extended toward the extracellular vestibule and complemented hydrophobic outer gate interactions, thereby further stabilizing the outward-open conformation and explaining its higher affinity to the three transporters relative to procaine and lidocaine (Figure 6).

A second critical feature of transporter binding involves interaction with a buried polar moiety at the TM3-TM8 interface (Li et al., 2024). Cognate substrates and several ligands engage this site either directly via the polar side chain of threonine, serine or asparagine, or via water-mediated hydrogen bonds (Please see these cryo-EM structures as examples: PDB-IDs: 7LIA, 8Y2D, 8Y95). Procaine possesses such a polar ring substituent, and docking placed it within hydrogen-bonding distance of Ser149 in DAT (Figure 7). In contrast, lidocaine lacks this functional group (Figure 1E), leaving a water molecule trapped in a partially hydrophobic environment which is an energetically unfavorable state that is consistent with its relatively weaker binding affinity. Although cocaine also lacks a polar ring substituent, its compact aromatic system (without the ortho-methyl present in lidocaine) permits a better fit, minimizing void volume and water occupancy. Furthermore, the rigid nitrogen-bridged aliphatic ring of cocaine elongates the molecule (Figure 1E), allowing its aromatic ring to insert more deeply within the binding pockets between the transmembrane helices, TM3 and TM8 (Figure 6).

## Discussion

Since its isolation in the mid-19th century, the pharmacological properties of cocaine have been extensively studied (Markel, 2011). Cocaine produces local anesthesia by blocking voltage-gated ion channels and exerts euphorigenic and addictive effects by inhibiting monoamine transporters. Its clinical use has been largely replaced by safer synthetic local anesthetics, limited topical applications persist (Hale et al., 2024); outside these niches, cocaine is primarily used illicitly.

Over recent decades, cocaine available on the illicit drug market in Vienna has commonly been adulterated (Kudlacek et al., 2017). The amount of adulteration is highly dynamic, with a tendency towards higher purity over the last decade (Magnolini et al., 2023). Our dataset also shows that adulterant profiles have shifted over time: levamisole, formerly predominant, has declined, while LAs have recently become more frequent adulterants.

Modern LAs were developed to retain cocaine’s local anesthetic efficacy while avoiding its psychoactive effects. Paradoxically, these same agents now appear regularly as cocaine adulterants. Several studies suggest that some LAs can influence central monoamine levels, a premise compatible with their demonstrated brain penetration in animals (Nakazono et al., 1991) and detection in human brain tissue from cocaine users (Knuth et al., 2018). Notably, Dorris and Gage (1980) reported dopamine elevation after ester-type LAs (e.g., procaine, tetracaine, propoxycaine), consistent with the behavior of inhibitors of DAT or amphetamine-like agents.

Subsequent work further supports a role for LAs in modulating monoaminergic signaling, although the findings are not entirely consistent. Ciarlone and Juras (1981) reported that lidocaine altered monoamine levels in multiple brain regions, whereas procaine increased serotonin and dopamine broadly across the brain as well as noradrenaline in selected areas. Sawaki and Kawaguchi (1989) likewise observed elevated dopamine and serotonin levels in the spinal cord following procaine administration, without changes in noradrenaline. In cell-based assays, Joyce et al. (2001) demonstrated that several LAs inhibited noradrenaline uptake in SH-SY5Y neuroblastoma cells, which endogenously express NET.

Our data only partially align with these findings: procaine inhibited DAT and NET, but not SERT at sub-millimolar concentrations, suggesting that procaine may increase extracellular dopamine and noradrenaline without directly influencing serotonin levels. The only systematic study of LAs at heterologously expressed transporters, by Sato and colleagues (2000), reported a similar profile, despite differing IC50 values for inhibitory potencies, with procaine showing DAT > NET > SERT selectivity and with no inhibition observed for lidocaine.

The frequently reported elevations of serotonin after procaine use despite absent SERT inhibition point to alternative mechanisms. To explore these, we examined the uptake-2 transporters OCT1–3 (SLC22A1–3), low affinity/high capacity polyspecific carriers expressed in the CNS and peripheral organs (Koepsell et al., 2007; Koepsell, 2020). All three OCTs are capable of transporting the monoamines serotonin, dopamine and noradrenaline to a certain extent (Koepsell et al., 2007). We previously found that cocaine inhibits OCT1 and OCT2, but not OCT3 (Angenoorth et al., 2021). Therefore, pharmacological alterations of these transporters would also influence serotonin homeostasis. Here, procaine inhibited OCT1 and OCT2 with a slightly lower IC_50_ compared to cocaine, while neither cocaine nor procaine substantially inhibited OCT3. Lidocaine showed only weak inhibition at OCT1 and OCT2, as the IC_50_ values are considerably higher than that of cocaine, and none at OCT3.

To summarize the inhibitory profile of our experiments: cocaine displayed a higher affinity for the three monoamine transporters of the SLC6 family than for the organic cation transporters of the SLC22 family, yielding the rank order: DAT = NET > SERT > OCT1 = OCT2 >> OCT3. In contrast, procaine favored OCT1 and OCT2, yielding: OCT1 > OCT2 = DAT > NET >> SERT = OCT3.

In the present study, we uncover procaine as another member of the “ester-type” LA family acting at organic cation transporters. Benzocaine, a third member of this family, mirrored procaine at SLC6 transporters in part—showing DAT inhibition with much lower potency at NET and no effect at SERT—but, unlike procaine, lacked inhibitory activity at OCT1–3. Dorris and Gage (1980) proposed that elevation of brain dopamine requires that a compound be a p-aminobenzoic acid (PABA) and exist as an ester of substituted ethanolamine. Among the LAs examined in this study, these criteria are met by procaine and benzocaine, but not by lidocaine.

To further explore these differences, we performed docking analyses to test whether procaine and lidocaine bind at DAT, NET, and SERT in distinct ways and to assess the stability of these interactions. Comparison of the ester-type LA with lidocaine, an amide-type LA, produced notable differences. Consistent with previous reports (Sato et al., 2000), lidocaine showed no effect on the three SLC6 transporters or on OCT3. In contrast, it inhibited OCT1 and OCT2, albeit with much lower potency than other LAs.

Docking analyses indicated that cocaine, through its methyl ester group, interacts with the hydrophobic outer gate, thereby stabilizing the outward-open conformation of the transporter and blocking substrate translocation. Procaine and lidocaine also fit within the binding pockets of the monoamine transporters; however, only procaine, owing to its polar ring substitution, formed a stable interaction with Ser149 in DAT, resulting in strong inhibition. In NET, the corresponding interaction involves Ala145 but is weaker due to greater distance. Lidocaine lacks this polar substitution of the ring, leading to less stable binding. Based on these observations, benzocaine would be expected to resemble procaine in its inhibitory profile—and indeed this was confirmed experimentally. These findings suggest that the presence of an amino group on the aromatic ring of an LA is critical for high-affinity inhibition of DAT.

Taken together, our findings indicate that although both ester- and amide-type LAs act on transporters of the SLC6 and SLC22 families, their selectivity is unlikely to reproduce a cocaine-like experience for the user because of a too low affinity. Their use as adulterants is more plausibly explained by their ability to block voltage-gated sodium channels to mimic the characteristic oral numbness of cocaine. Nevertheless, the addition of procaine, benzocaine, or lidocaine to illicit cocaine cannot be regarded as irrelevant or harmless to consumers. In particular, the relatively high-affinity inhibition of OCT1 and OCT2—transporters with widespread central and peripheral expression (Koepsell, 2020)—raises the likelihood of adverse events. In addition OCTs are responsible for the transport of a wide range of substances. (Koepsell, 2020). The concern is amplified by the finding that, in some cases, procaine content exceeds that of cocaine itself, increasing the risk of side effects mediated by blockade of OCT1-and OCT2. Routine analysis of illicitly sourced cocaine and clear communication of the risks associated with adulterants therefore remain essential for reducing drug-related harm and fatalities.

## Limitations

Our conclusions are based on heterologous expression systems and radiotracer uptake assays; *in vivo* concentrations at transporter sites after street level co exposure are uncertain. Docking provides qualitative hypotheses but lacks explicit solvent dynamics and full conformational sampling. Finally, mixture effects among multiple adulterants (and with cocaine) were not modeled. Future work should quantify brain/plasma exposures of specific LAs in typical cutting ratios and assess combined effects under physiologic ionic conditions and firing patterns.

## Data Availability

The original contributions presented in the study are included in the article/supplementary material, further inquiries can be directed to the corresponding author.

## Footnotes

1
https://www.unodc.org/documents/data-and-analysis/WDR_2025/WDR25_B1_Key_findings.pdf
